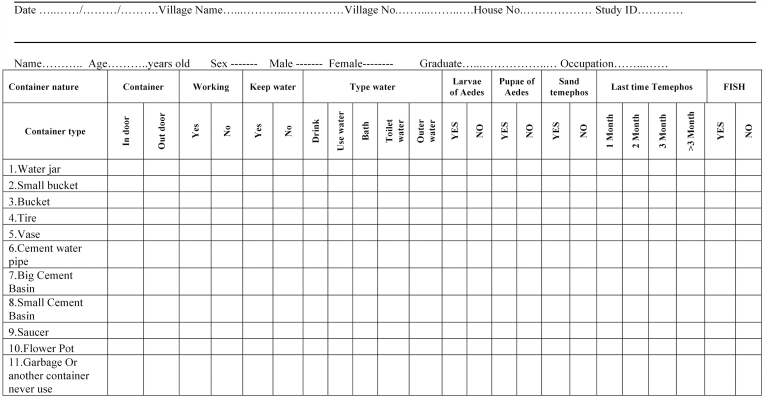# Effectiveness of dengue training programmes on prevention and control among high school students in the Yangon region, Myanmar

**DOI:** 10.1016/j.heliyon.2023.e16759

**Published:** 2023-05-27

**Authors:** Soe Htet Aung, Suparat Phuanukoonnon, Aye Mon Mon Kyaw, Saranath Lawpoolsri, Patchara Sriwichai, Ngamphol Soonthornworasiri, Podjanee Jittamala

**Affiliations:** a Department of Tropical Hygiene, Faculty of Tropical Medicine, Mahidol University, Thailand; b Department of Social and Environmental Science, Faculty of Tropical Medicine, Mahidol University, Thailand; c Central Epidemiology Unit, Department of Public Health, Ministry of Health, Myanmar; d Department of Medical Entomology, Faculty of Tropical Medicine, Mahidol University, Thailand

**Keywords:** Dengue school training programme, Knowledge, Attitude and practices, Yangon, Myanmar

## Abstract

**Background:**

Dengue is one of the health problems in Myanmar. Thus, health promotion in schools is considered a key approach for reducing risk-taking behaviours related to dengue.

**Objectives:**

The study aimed to evaluate a dengue training programme for high school students to measure changes in knowledge, attitude and practices (KAP) towards dengue; evaluate the effectiveness of the programme in improving prevention and control practices among families and determining changes in larval indices in their dwelling places.

**Methodology:**

The dengue school training programme was conducted for Grades 9 and 10 students in Yangon. In total, 300 students in the intervention school received training and were compared with 300 students as control. KAP was assessed using a self-administered questionnaire, whereas larval and control practice surveys were conducted at the homes of both groups 3 months before and after the programme.

**Results:**

The KAP scores of the intervention group increased after the programme. Moreover, the programme improved prevention and control practices and decreased the larval indices in the intervention group. Students from the same group with high scores in knowledge and self-reported practices were less likely to exhibit *Aedes* larval positivity in their residential areas.

**Conclusion:**

This study demonstrated the impact of the dengue training programme on the KAP of students and short-term family larval control practices, which influenced household larval indices.

## 1 Introduction

Dengue affects more than one-third of the world population [1], with 3.9 billion people living in dengue-endemic areas. The global incidence of dengue fever has approximately doubled in the past three decades and is expected to increase in Asia, sub-Saharan Africa and Latin America [2]. Approximately 50% of the world's population lives in dengue risk areas [3].

The dengue virus belongs to the *Flaviviridae* family with four serotypes (i.e. DEN-1, DEN-2, DEN-3 and DEN-4). *Aedes* is the main culprit of dengue, while the primary vector is Aedes aegypti [4]. The clinical manifestations of dengue are wide-ranging from flu-like symptoms to severe conditions, such as dengue shock syndrome and dengue haemorrhagic fever. The fatality rate of severe dengue can exceed 20% without proper treatment. The current efforts on dengue prevention are focused on vector control [5].

The incidence of dengue in South East Asia (SEA) has gradually increased owing to demographic changes and ecological disruption [6,7]. In particular, the incidence from 2015 to 2019 dramatically increased by 46%, and 1.3 billion people live in dengue endemic areas in 10 SEA countries [8]. Notably, the high productivity costs associated with dengue episodes have led to considerable financial burden in SEA [9].

Dengue was first recognised in Myanmar during the 1960s. Specifically, the first dengue outbreak in Yangon occurred in 1974. In 2015, the prevalence rates of dengue in all the regions were reported to be high, whereas areas with the highest mortality rates due to dengue were Yangon, Sagaing and Ayawaddy [10]. Yangon is the largest city in Myanmar [11] and holds the highest incidence rates of dengue from 2009 to 2018 [12]. Dengue surveillance was introduced in 1964, and since then, the National Committee on Dengue and the *Aedes* Mosquito Control Unit were established. Moreover, the current national dengue control strategies were adopted in line with the Asian Pacific Regional Dengue Strategic Plan. The primary objective of these strategies was reducing the incidence of dengue. The integrated vector control is a cornerstone of dengue prevention and control measure. At the local level, the commonly used strategy was community- and school-based dengue prevention and control programmes [13].

Health promotion focuses on the reduction of risk-taking and the increase of protective behaviours. Schools are ideal settings for learning disease prevention and health promotion [14]. Since 2006, the Ministry of Health (MOH) has launched dengue health education in schools. However, as of 2015, it covered only 38.8% of schools [15]. Community members exhibited the positive impact of health education by conducting prevention and control practices [16]. Thus, our study infers that school-based dengue control programmes increased the knowledge of students, which may influence behaviours related to dengue control practices in their homes [17]. Several studies have revealed that the health-related curriculum of schools provided insufficient knowledge about dengue control methods that could be translated into appropriate practices in communities [[18], [19], [20]]. There are limited educational interventions in Myanmar; therefore, an effective school training programme on household larval control is warranted in this country. Thus, the current study aimed to assess the knowledge, attitude and practice of students regarding dengue and explore the effectiveness of the dengue training programme and household larval control practices.

## 2 Material and methods

### 2.1 Study design

This study is quasi-experimental intervention study on Grades 9 and 10 students from two high schools in Yangon, Myanmar. The study provided the dengue training programme to students in the intervention group after a pre-test and conducted a post-test after 3 months. In the control group, the students received only the routine health education programme provided by the school and local health sectors. The students in the intervention groups conducted a survey on larval indices at their dwelling places under the close supervision of the investigators and entomologists 3 months before and after the training. However, the control group the larval survey was performed by the researcher teams.

The study was conducted from August 2018 to April 2019. Two townships in the Yangon region (Hmawbi and Kaw Hmu) were selected based on their similarity in larval indices. The distance between these two townships was 80 km. The schools were randomly selected as the intervention or control school. A total of 300 students in Grades 9 and 10 from each high school were recruited. Written informed consent was provided by parents, while assent by students was obtained prior to the study.

### 2.2 Sample size

The sample size was calculated using the two independent proportions formula. The anticipated level of knowledge of the control group was derived from Suwanbamrung et al. [21], i.e. on the basic dengue knowledge questionnaire with regard to dengue: 70.9% (p1). The anticipated proportion of students with knowledge in the study group was 81% (p2). With a test power of 80% and a significance level at 5%, the required minimal sample for this study is 280. Correction for drop-out was set at 10% such that a sample size of approximately 300 per group is considered appropriate.

### 2.3 Interventions

**Intervention**: Dengue training programme was applied to the intervention group.

**Tool for training**: The main content included the biology and mode of transmission of *Aedes*, common clinical manifestations and prevention and control methods. Visual aids, such as flipcharts, audio–visual media and video, were used. The students received intensive training by entomologists before performing larval survey and using the survey form under the supervision of entomologists, investigators and school health teachers (i.e. teaching and hands-on training). This training focused on the use of the larval survey methods in the residential areas of students, including the identification of breeding sites and methods for controlling the vector (e.g. using temephos or larvivorous fish or cleaning and covering water containers), identifying of the mosquito life cycle and calculation of the index.

**Training method**: Actual training was performed by classifying the students into three subgroups with 100 students in each group. The average time for in-class training, including discussion and question-and-answer sessions, was 2–3 h per group. Training materials, such as leaflets and infographics on dengue, were distributed to the students to share with their families (Supplementary Information 2).

**KAP testing:** Pre- and post-test questionnaire was used to measure KAP. Pre-test was performed 2 weeks before training, whereas post-test was performed 3 months after school training in the intervention group. In the control group, the pre- and post-tests were performed at an interval of 3 months without training program. **Tool for KAP testing:** The questionnaire was drafted following the literature review. It comprised demographics and knowledge, attitude and practices (KAP) regarding dengue transmission, infection, prevention and control. The number of questions on KAP differed; thus, the ranges of the total scores also differed. Knowledge covers 21 questions on transmission, breeding sites, common features of the disease and prevention and control. A correct response takes a score of 1; otherwise, it takes a value of 0. The scores were summed and categorised into three groups based on the modified Bloom cut-off point [22]; scores of ≥80% (18–21 points), 60–80% (12–17 points) and ≤59% (≤11 points) were considered good, moderate and poor levels of knowledge, respectively. Attitude covers 12 questions, including attitude towards the harm/threat of the disease, personal protective measures, preventive measures, larval controls and existing health promotion programme. Items were rated on a 4-point Likert-type scale—from 4 = Strongly agree to 1 = Strongly disagree for positive statements regarding attitude. Inversely, the scores were opposite for negative statements. The scores for attitude were summed and categorised into three groups: positive (>80%; 41–48), neutral [[23], [24], [25], [26], [27], [28], [29], [30], [31], [32], [33], [34], [35]] and negative [[12], [13], [14], [15], [16], [17], [18], [19], [20], [21], [22], [36], [37], [38], [39], [40]]. Lastly, practices presented nine questions using dummy variables, where ‘Yes’ takes a value of 1 and ‘No’ takes a value of 0. Scores for practice were summed and categorised into three groups: good (8–9), fair (6–7) and poor (0–5).

The questionnaire was tested for internal consistency among 60 students, whose data were excluded from the final analysis. Cronbach's alpha reached 0.7.

The larval survey method: The larval survey primarily comprised the identification of breeding sites, characteristics of containers, covering methods, frequency and methods for cleaning, use of temaphos and identifying the presentation of *Aedes* larva using the larval survey form. As per group, the intervention group conducted the larval survey under the supervision of the entomologists and researcher two weeks before and 3 months after the training. However, the larval survey was performed by the research teams (Fig. 1).

**Fig. 1:**
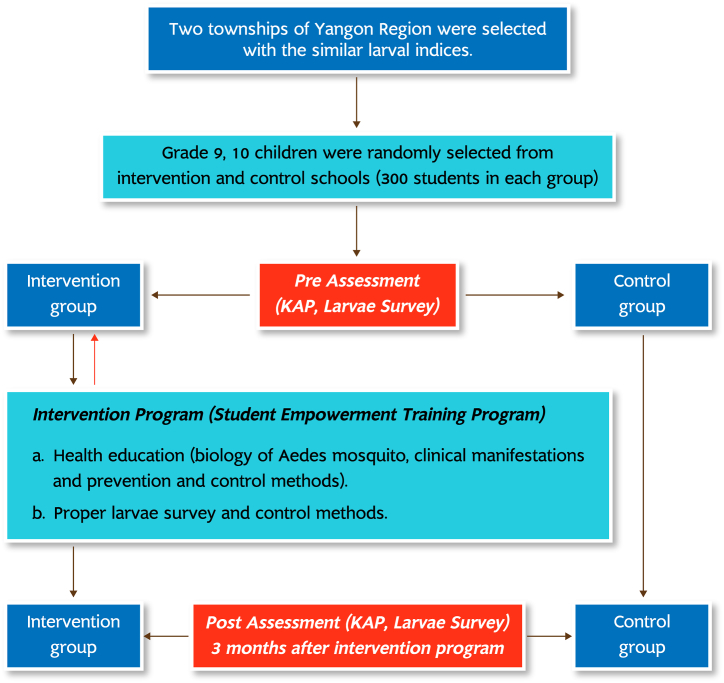
Flow of the study.

Tool for larval survey: The Department of Entomology, Department of Tropical Environment and Social Science and Department of Tropical Hygiene, Faculty of Tropical Medicine, Mahidol University (Supplementary Information 1) developed a survey checklist for the larval survey.

The effectiveness of the training was evaluated through changes in larval control practices (e.g. use of temephos, weekly cleaning and properly covering water containers) and in larval indices (House Index [HI], Breteau Index [BI] and Container Index [CI]), moreover, the study explored the correlation between the KAP scores and detection of larva (larval positivity) for both groups (Fig. 1).

The Faculty of Tropical Medicine (MUTM 2019-025-01) and Defence Services Research Centre (IRB/2018/26) approved the study. The protocol for school intervention in schools was approved by the Township Education Department of the respective townships and school principals. Further, the study was registered under the Thai Clinical Trials Registry (TCTR20200308002).

### 2.4 Statistical analysis

Data on KAP and the larval survey were coded, cleaned, reviewed and entered into Microsoft Assess (2016). Statistical analysis was conducted using STATA version 14. Additionally, descriptive statistics was performed to obtain the mean, standard deviation, frequencies and percentages. For categorical variables, data were reported as proportions (%) and compared using chi-squared tests. Multiple logistic regression analysis was used to identify the factors associated with each KAP level and *Aedes* larval positivity at participant homes. *P*-value <0.05 was considered statistically significant.

## 3 Results

### 3.1 Socio-demographic characteristics

Both schools were under the government. The male and female student ratio and the mean age were similar for both groups. More than half of the students came from middle-income families, with Bamar as their main ethnicity. The housing style for both groups was similar. Lastly, the majority of living places had electricity and toilets (Table 1).

**Table 1:** Socio-demographic characteristics of the participants.

	Intervention *n* (%)	Control *n* (%)	*P value*
Age (mean ± SD)	14.32 (±1.2)	14.74 (±1.6)	0.512
**Gender**			
Male	133 (44.3)	140 (46.7)	0.566
Female	167 (55.7)	160 (53.3)	
**Education**			
Grade 9	143 (47.7)	147 (49.0)	0.744
Grade 10	157 (52.3)	153 (51.0)	
**Income**			
100,000–300,000 Kyats	84 (28.0)	99 (33.0)	0.413
300,000–500,000 Kyats	176 (58.7)	164 (54.7)	
> 500,000 0 Kyats	40 (13.3)	37 (12.3)	
**Ethnicity**			
Bamar	207 (69.0)	230 (76.7)	0.199
Kayin	17 (5.7)	14 (4.7)	
Shan	28 (9.3)	19 (6.3)	
Others	48 (16.0)	37 (12.3)	
**Toilet in dwelling place**			
Own toilet	291 (97.0)	295 (98.6)	0.279
Shared Toilet	9 (3.0)	5 (1.4)	
**Electricity in dwelling place**			
Present	285 (95.0)	280 (93.3)	0.384
Absent	15 (5.0)	20 (6.7)	

### 3.2 Knowledge on dengue and its prevention and control

Table 2 presents the distribution of the correct responses of students in both groups before and after training. In the pre-test, both groups correctly answered that the major vector of dengue was *Aedes*, which bite primarily during the daytime (*p* > 0.05). After training, significant improvements were noted in both topics only for the intervention group (*p* < 0.001). Regarding breeding site, correct responses to common outdoor breeding sites (i.e. discarded tyres, garbage and water tanks) were similar in the pre-test for both groups but significantly increased for the intervention group in the post-test. Prior to intervention, the majority of students misunderstood that unlikely outdoor breeding sites (i.e. wastewater drainage and rice fields) were common breeding sites of *Aedes*. After the training, however, >95% of the students in the intervention group responded that dirty water in the drainage was not a breeding site, whereas 1/3 of both groups continued to believe that rice fields are breeding sites.

**Table 2:** Scores of both groups for knowledge before and after the intervention.

No.	Questions		Intervention Group *n* (%)			Control Group n (%)		
			Before	After	*P* - value	Before	After	*P - value*
1	Dengue is caused by bite of *Aedes* mosquito		216 (72.0)	296 (98.6)	<**0.001**	216 (72.0)	263 **(87.6)	<**0.001**
2	*Aedes* mosquitoe bites primarily during the day		195 (65.0)	293 (97.6)	<**0.001**	190 (63.3)	257**(85.6)	<**0.001**
3	Outdoor mosquito breeding site	discarded tire	250 (83.3)	295 (99.0)	<**0.001**	242 (80.6)	247** (82.3)	0.599
		garbage	256 (85.3)	298 (99.3)	<**0.001**	246 (82.0)	250**(83.3)	0.666
		water tank	240 (80.0)	295 (98.3)	<**0.001**	264^#^ (88.0)	252 **(84.0)	0.158
		rice field (no)^€^	114 (38.0)	196 (65.3)	<**0.001**	138^#^ (46.0)	151**(50.3)	<**0.001**
		waste water drain (no)^€^	57 (19.0)	287 (95.6)	<**0.001**	54 (18.0)	50 **(16.6)	0.666
4	Indoor mosquito breeding site	water containers for flushing toilet	229 (76.3)	297 (99.0)	<**0.001**	248^#^ (82.6)	254**(84.6)	0.508
		water container for daily use	195 (65.0)	297 (99.0)	<0.001	187 (62.3)	210**(70.0)	0.047
		saucers for food cupboard stand	217 (72.3)	298 (99.3)	<**0.001**	216 (72.0)	241**(80.3)	0.017
5	Major symptom of Dengue	fever	274 (91.3)	298 (99.3)	<**0.001**	291^#^ (97.0)	284 **(94.6)	0.153
		skin rash	232 (77.3)	294 (98.0)	<**0.001**	253^#^(84.3)	242 **(80.6)	0.237
		bleeding	79 (26.3)	280 (93.3)	<**0.001**	68 (22.6)	94 ** (31.3)	0.017
6	Cover water container with lid		269 (89.6)	298 (99.3)	<**0.001**	267 (89.0)	268 **(89.3)	0.895
7	Weekly changing stored water		265 (88.3)	297 (99.0)	<**0.001**	254 (84.6)	283*(94.3)	<**0.001**
8	Release larvivorous fish in water container		184 (61.3)	293 (97.6)	<**0.001**	159^#^(53.0)	166**(55.3)	0.566
9	Use of temephos in water		175 (58.3)	263 (87.6)	<**0.001**	187 (62.3)	205**(68.3)	0.123
10	Use of mosquito net during the day nap		259 (86.3)	297 (99.0)	<**0.001**	261 (87.0)	286 **(95.3)	< **0.001**
11	Wear the colorful long sleeves and pants		243 (81.0)	284 (94.6)	<**0.001**	219^#^(73.0)	229**(76.3)	0.348
12	Dengue may cause death		255 (85.0)	295 (98.3)	<**0.001**	263 (87.6)	243**(81.0)	0.025
13	Dengue is preventable disease		240 (80.0)	293 (97.6)	<**0.001**	260^#^(86.6)	259 **(86.3)	0.905

€ The correct answer is “No”.

^#^*P*-value <0.05.

^##^*P*-value <0.001 between the two groups before study intervention.

*P-value <0.05 ** *P*-value <0.001 between the two groups after study intervention.

For common indoor breeding sites, a similar number of students correctly answered in the pre-test that water containers for daily usage and for toilet flushing and water cup for legs of cupboard (to prevent the food from ants) were breeding sites. After the training, the number of students in the intervention group who provided correct answers significantly increased compared with the control group (*p* < 0.001). More students in the control group correctly answered that fever was a major symptom of dengue compared with the intervention group in the pre-test (*p* = 0.003). However, both groups exhibited similar levels of knowledge about skin rash and bleeding as clinical manifestations in the pre-test (*p* > 0.05). The students of both groups were least concerned about bleeding as a clinical feature of the disease prior to the training. In the post-test, the intervention group displayed a significant increase in correct responses to the three common clinical features of dengue, including bleeding manifestation.

Baseline knowledge about prevention and control was similar for both groups. The baseline correct responses to using temephos in water and using larvivorous fish were low for both groups prior to training. Moreover, both groups displayed similar background knowledge about the threat of dengue (e.g. ‘Dengue fever is a preventable disease’ (*p* = 0.028) and ‘Dengue may cause death’ (*p* = 0.342).

After training, the students in the intervention group exhibited significant improvements in knowledge for all items including biology, transmission, preventive measures and knowledge about the disease. Interestingly, the correct responses of the students in control group also significantly increased, especially in knowledge about preventive and control (‘changing stored water on a weekly basis’ (*p* < 0.001) and ‘using a mosquito net during the day’ (*p* < 0.001) (Table 2).

### 3.3 Attitude towards dengue and its prevention and control

More than half of the students in both groups similarly strongly agreed/agreed to the statement that ‘dengue can occur all year round in Yangon’ (*p* = 0.274). Regarding repeated infection and death, the perception of the students in the intervention group significantly increased as evidenced by their responses to the statements ‘Previous dengue patients can get sick again with dengue’ and ‘DHF may lead to death if a prompt and proper treatment is not given’ in the post-test (*p* < 0.001).

Regarding attitude towards control methods, most students in both groups strongly agreed/agreed to the statements ‘A weekly cleaning of mosquito breeding sites around the house is essential’ and ‘Covering water containers will prevent mosquitoes from laying eggs’. After training, the ratios of agreement to disagreement increased significantly for the intervention group (*p* < 0.001).

For negative statements, more than one-third of the students in both groups strongly agreed/agreed to the statement ‘Using temephos in water could be harmful to health’ (*p* = 0.124), whereas approximately two-third of the students strongly agreed/agreed with the items ‘Regularly monitoring mosquito breeding sites in the home is difficult’ (*p* = 0.207) and ‘Wearing protective clothing during daytime is very hot and inconvenient’ (*p* = 0.360) in the pre-test. After training, the number of strongly disagree/disagree responses increased significantly for the intervention group (*p* < 0.001). For the item ‘Sleeping under mosquito net during the day is inconvenient’, more students in the intervention group strongly agreed/agreed than those in the control group in the pre-test (*p* < 0.006). After training, the majority of the students in the intervention group exhibited disagreement towards this negative statement on this preventive measure (*p* < 0.001), whereas the attitude of the control group remained unchanged (*p* = 0.207).

Prior to training, students from both groups similarly strongly agreed/agreed with the item ‘Mosquito coils emit a bad smell and can be harmful to health.’ However, they exhibited a higher degree of agreement with the unfavourable smell and the harmful effects of the chemical substances in mosquito coils after the training.

For regular health promotion from the health department, approximately 70% of the students strongly agreed/agreed that health promotion was effective (*p* = 0.046) before the training. Afterwards, only the students in the intervention group exhibited increased confidence in the existing training programmes conducted by the relevant authorities (*p* < 0.001; Table 3).

**Table 3:** Scores for both groups for attitude before and after the intervention.

No.	Questions		Intervention Group *n* (%)			Control Group *n* (%)		
			Before	After	*P -value*	Before	After	*P -value*
1	Dengue fever can occur throughout all year round in Yangon	Strongly Agree	75 (25.0)	188 (62.6)	<**0.001**	82 (27.3)	75 (25.0)	0.242
		Agree	105 (35.0)	101 (33.6)		111 (37.0)	94 (31.3)	
		Disagree	83 (27.6)	8 (2.6)		70 (23.3)	84 (28.0)	
		Strongly Disagree	37 (12.3)	3 (1.0)		37 (12.3)	47 (15.6)	
2	DHF may lead to death if a prompt and proper treatment not given.	Strongly Agree	108 (36.0)	170 (56.6)	<**0.001**	118 (39.3)	104 (34.6)	0.143
		Agree	139 (46.3)	127 (42.3)		142 (47.3)	138 (46.0)	
		Disagree	44 (14.6)	2 (0.6)		31 (10.3)	39 (13.0)	
		Strongly Disagree	9 (3.0)	1 (0.3)		9 (3.0)	19 (6.3)	
3	Previous dengue patient can get sick again with dengue.	Strongly Agree	144 (48.0)	205 (68.3)	<**0.001**	153 (51.0)	159 (53.0)	0.657
		Agree	110 (36.6)	82 (27.3)		99 (33.0)	88 (29.3)	
		Disagree	22 (7.3)	10 (3.3)		30 (10.0)	37 (12.3)	
		Strongly Disagree	24 (8.0)	3 (1.0)		18 (6.0)	16 (5.3)	
4	Weekly cleaning the mosquito breeding site around the house is essential.	Strongly Agree	157 (52.3)	219 (73.0)	<**0.001**	160 (53.3)	180 (60.0)	0.106
		Agree	128 (42.6)	75 (25.0)		130 (43.3)	103 (34.3)	
		Disagree	6 (2.0)	4 (1.3)		6 (2.0)	10 (3.3)	
		Strongly Disagree	9 (3.0)	2 (0.6)		4 (1.3)	7 (2.3)	
5	Cover the water container will prevent mosquito laying eggs.	Strongly Agree	119 (39.6)	202 (67.3)	< **0.001**	138 (46.0)	150 (50.0)	0.109
		Agree	152 (50.6)	93 (31.0)		140 (46.6)	119 (39.6)	
		Disagree	20 (6.6)	2 (0.6)		16 (5.3)	16 (5.3)	
		Strongly Disagree	9 (3.0)	3 (1.0)		6 (2.0)	15 (5.0)	
6	Regularly Changing water in the flower vases is inconvenient. (Negative statement)	Strongly Agree	36 (12.0)	9 (3.0)	<**0.001**	42 (14.0)	49 (16.3)	0.253
		Agree	36 (12.0)	25 (8.3)		41 (13.6)	54 (18.0)	
		Disagree	101 (33.6)	140 (46.6)		102 (34.0)	84 (28.0)	
		Strongly Disagree	127 (42.3)	126 (42.0)		115 (38.3)	113 (37.6)	
7	Using temephos in the water can be harmful to health (Negative Statement)	Strongly Agree	27 (9.0)	3 (1.0)	<**0.001**	26 (8.6)	41 (13.6)	0.011
		Agree	88 (29.3)	11 (3.6)		71 (23.6)	74 (24.6)	
		Disagree	116 (38.6)	138 (46.0)		132 (44.0)	96 (32.0)	
		Strongly Disagree	69 (23.0)	148 (49.3)		71 (23.6)	89 (29.6)	
8	Regularly check and eliminate the mosquito breeding site in living area is not easy to do (Negative Statement)	Strongly Agree	50 (16.6)	15 (5.0)	<**0.001**	49 (16.3)	57 (19.0)	0.014
		Agree	129 (43.0)	32 (10.6)		145 (48.3)	106 (35.3)	
		Disagree	80 (26.6)	129 (43.0)		64 (21.3)	81 (27.0)	
		Strongly Disagree	41 (13.6)	124 (41.3)		42 (14.0)	56 (18.6)	
9	Mosquito coil emit bad smell smoke that harmful to health	Strongly Agree	142 (47.3)	237 (79.0)	<**0.001**	145 (48.3)	151 (50.3)	0.130
		Agree	135 (45.0)	35 (11.6)		136 (45.3)	117 (39.0)	
		Disagree	18 (6.0)	4 (1.3)		12 (4.0)	16 (5.3)	
		Strongly Disagree	5 (1.6)	24 (8.0)		7 (2.3)	16 (5.3)	
10	Wearing the colorful long sleeves and pants in daytime is very hot and inconvenient (Negative Statement)	Strongly Agree	43 (14.3)	14 (4.6)	<**0.001**	65 (21.6)	60 (20.0)	0.949
		Agree	141 (47.0)	42 (14.0)		108 (36.0)	109 (36.3)	
		Disagree	73 (24.3)	164 (54.6)		86 (28.6)	91 (30.3)	
		Strongly Disagree	43 (14.3)	80 (26.6)		41 (13.6)	40 (13.3)	
11	Sleeping in the mosquito net during the day time nap is inconvenient (Negative Statement)	Strongly Agree	27 (9.0)	16 (5.3)	<**0.001**	32 (10.6)	42 (14.0)	0.207
		Agree	91 (30.3)	15 (5.0)		73 (24.3)	57 (19.0)	
		Disagree	110 (36.6)	167 (55.6)		119 (39.6)	112 (37.3)	
		Strongly Disagree	72 (24.0)	102 (34.0)		76 (25.3)	89 (29.6)	
12	Health promotion programs from health department and schools are effective	Strongly Agree	58 (19.3)	141 (47.0)	<**0.001**	60 (20.0)	88 (29.3)	0.313
		Agree	146 (48.6)	145 (48.3)		166 (55.3)	141 (47.0)	
		Disagree	76 (25.3)	11 (3.6)		59 (19.6)	54 (18.0)	
		Strongly Disagree	20 (6.6)	3 (1.0)		15 (5.0)	17 (5.6)	

### 3.4 Self-reported practice regarding dengue prevention and control

Table 4 summarises the self-reported practices of the students. Both groups displayed similar self-reported practices for all items. However, the control group reported a significantly high percentage of practices for ‘weekly changing of water in pot trays’ (*p* < 0.001). The number of students who reported the use of temephos in water was low for both groups at baseline (intervention = 173/300, 57.6%; control = 165/300, 55.0%; *p* = 0.510). After training, the percentage of students with self-reported practices significantly increased for the intervention group (*p* < 0.001). Interestingly, the use of temephos in water remained low after training in the intervention group (pre-training = 173/300, 57.6%; post-training = 203/300, 67.6%; Table 4).

**Table 4:** Scores for both groups in self-reported practice regarding dengue prevention and control before and after the intervention.

No	Variables	Intervention Group *n* (%)			Control Group *n* (%)		
		Before	After	*P-value*	Before	After	*P-value*
1	Weekly changing the water in the pot tray.	261 (87.0)	300 (100.0)	<**0.001**	296^##^ (98.6)	288** (96.0)	0.043
2	Weekly changing the water containers under the fridge.	255 (85.0)	295 (98.3)	<**0.001**	260 (86.6)	262** (87.3)	0.808
3	Covering water containers properly both inside and outside the house.	266 (88.6)	299 (99.6)	<**0.001**	279 (93.0)	273** (91.0)	0.367
4	Cleaning the bushes and drainage system in the dwelling environment.	283 (94.3)	299 (99.6)	<**0.001**	281 (93.6)	277** (92.3)	0.522
5	Flip, burned, discarded the unused potential mosquito breeding site/container in the house.	241 (80.3)	285 (95.0)	<**0.001**	241 (80.3)	251** (83.6)	0.288
6	Using temephos in water containers.	173 (57.6)	203 (67.6)	0.011	165 (55.0)	180** (60.0)	0.215
7	Using insecticide spray in the house	217 (72.3)	259 (86.3)	<**0.001**	230 (76.6)	238* (79.3)	0.430
8	Sleeping in the mosquito net during the daytime nap.	291 (97)	300 (100.0)	**0.003**	278^#^ (92.6)	288* (96.0)	0.077
9	Wearing the colorful long sleeves shirt and pants during daytime.	258 (86.0)	298 (98.3)	<**0.001**	260 (86.6)	284* (94.6)	0.001

^#^*P-*value <0.05.

^##^*P*-value <0.001 between the two groups before study intervention.

*P-value <0.05 ** *P*-value <0.001 between the two groups after study intervention.

### 3.5 Levels of knowledge, attitude and self-reported practices of both groups before and after intervention

Table 5 presents the level of KAP before and after training. KAP scores were categorised into three groups by using the modified Bloom cut-off point [23]. The KAP levels were similar for both groups in the pre-test. Most students exhibited a moderate level of knowledge, neutral attitude and good self-reported practice. After the training, however, the KAP levels for the intervention group significantly increased (*p* < 0.001; Table 5).

**Table 5:** Level of knowledge, attitude and self-reported practices of both groups before and after intervention.

		Before			After		
		Intervention Group *n* (%)	Control Group *n* (%)	*P -value*	Intervention Group *n* (%)	Control Group *n* (%)	*P* -value
KNOWLEDGE (21) * (1–21)^#^	High Knowledge (18–21 scores)	71 (23.6)	78 (26.0)	0.803	256 (85.0)	80 (26.6)	<0.001
	Moderate Knowledge (12-17scores)	183 (61.0)	177 (59.0)		25 (8.3)	193 (64.3)	
	Low Knowledge (1-11scores)	46 (15.3)	45 (15.0)		19 (6.3)	27 (9.0)	
ATTITUDE (12) * (12–48)^#^	Positive attitude (41–48 scores)	36 (12.0)	43 (14.3)	0.673	175 (58.3)	34 (11.3)	<**0.001**
	Neutral Attitude (28–40 scores)	253 (84.3)	245 (81.6)		118 (39.3)	256 (85.3)	
	Negative Attitude (12–27 scores)	11 (3.6)	12 (4.0)		7 (2.3)	10 (3.3)	
PRACTICES (9) * (1–9)^#^	Good self- reported practice (8–9 Scores)	173 (57.6)	175 (58.3)	0.985	253 (84.3)	222 (74.0)	<**0.001**
	Fair self-reported practice (6–7 Scores)	84 (28.0)	83 (27.6)		34 (11.3)	56 (18.6)	
	Poor self-reported practice (1–5 Scores)	43 (14.3)	42 (14.0)		13 (4.3)	22 (7.3)	

### 3.6 Effectiveness of the school training programme

The study evaluated the effectiveness of the dengue training programme using changes in actual practices, which were collected during the vector survey, changes in larval indices and the association between KAP and larval positivity in the household.

### 3.7 Changes in larval control practices

The larval survey included measurable practices, such as the use of temephos, weekly cleaning of water containers and properly covering water containers. Before the intervention, the number of water containers was 1662 and 2234 for the intervention and control groups, respectively. The number of water containers slightly decreased to 1612 and 2153 at 3 months after training. The rates of observed practices by family members were relatively lower than those of the self-reported practices of the students. The larval control practices between the two groups were similar before the intervention. In the second survey, significant improvements were noted for ‘weekly cleaning of water containers’ and ‘properly covering water containers’ only for the intervention group (*p* < 0.001), whereas no difference was observed in the use of temephos for both groups in pre- and post-tests (Table 6). Lastly, the larval survey did not observe the use of larvivorous fish for both groups.

**Table 6:** Changes in larval control practice after dengue school training program.

Practice	Before			After		
	Intervention group (N = 1662) *n* (%)	Control group (N = 2234) *n* (%)	*P*-value	Intervention group (N = 1612) n (%)	Control group (N = 2153) *n* (%)	*P*-value
Use of temephos	925 (55.6)	1272 (56.9)	0.425	948 (58.8)	1251 (58.1)	0.665
Cleaning water container weekly	592 (35.6)	772 (34.5)	0.492	880 (54.5)	729 (33.8)	<0.001
Covering water container appropriately	696 (41.8)	920 (41.1)	0.663	905 (56.1)	946 (43.9)	<0.001

### 3.8 Change in household larval indices

The larval indices of the intervention and control groups decreased after training (Table 7).

**Table 7:** Household larval indices before and after intervention for both groups.

No	Index	Intervention group *n* (%)			Control group *n* (%)		
		Before	After	*P*-value	Before	After	*P-*value
1	House index (HI)	36.6%	7.6%	<0.001	31.0%	17.0%	0.02
2	Container index (CI)	11.4%	3.5%	0.033	8.3%	5.0%	0.349
3	Breteau index (BI)	63.3%	19.0%	<0.001	62.3%	36.0%	<0.001

### 3.9 Association between KAP and larval positivity in the household for both groups

Multivariate analysis employed three levels of each KAP domain to determine the association between KAP levels and larval positivity. The high level of knowledge of the intervention group was associated with less chances to detect *Aedes* larvae in their homes [AOR (95% CI): 0.10 (0.08–0.42), *p* < 0.001]. Positive self-reported practices in the intervention group were correlated with low levels of larval positivity [AOR (95% CI): 0.07 (0.03–0.20), *p* < 0.001], whereas no strong correlation, such as that observed in knowledge and practice domains, was observed between positive attitude and larval positivity [AOR (95% CI): 0.45 (0.15–0.71), *p* = 0.01; Table 8].

**Table 8:** KAP variables associated with larval positivity after the dengue school training program.

		*n*	Intervention group				*n*	Control group			
			Univariate		Multivariate			Univariate		Multivariate	
			COR (95% CI)	*P-*value	AOR (95% CI)	*P-*value		COR (95% CI)	*P*-value	AOR (95% CI)	*P-*value
Knowledge	Low knowledge	19	**Ref**		**Ref**		27	**Ref**		**Ref**	
	Moderate knowledge	25	0.5 (0.04–0.81)	0.052	0.30 (0.02–0.72)	0.023	193	2.26 (1.22–4.19)	0.009	2.51 (1.19–5.27)	0.015
	High knowledge	256	0.2 (0.08–0.51)	<0.001	**0.1** (0.08–0.42)	<**0.001**	80	1.12 (0.44–2.84)	0.803	1.08 (0.41–2.85)	0.775
Attitude	Negative attitude	7	**Ref**		**Ref**		10	**Ref**		**Ref**	
	Neutral attitude	118	0.27 (0.11–0.65)	0.003	0.40 (0.14–1.09)	0.076	256	2.25 (1.16–4.39)	0.016	2.85 (1.30–6.26)	0.009
	Positive attitude	175	0.33 (0.14–0.77)	0.010	0.45 (0.15–0.71)	0.011	34	0.42 (0.22–0.78)	0.006	0.38 (0.18–0.79)	0.010
Practices	Poor practice	13	**Ref**		**Ref**		22	**Ref**		**Ref**	
	Fair practice	34	0.17 (0.05–0.51)	0.002	0.25 (0.07–0.81)	0.021	56	0.80 (0.41–1.57)	0.529	0.93 (0.46–1.89)	0.854
	Good practice	253	0.06 (0.02–0.15)	<0.001	**0.07** (0.03–0.20)	<**0.001**	222	0.23 (0.05–1.02)	0.054	0.29 (0.06–1.42)	0.129

Cordially, = Crude Odds Ratio, AOR = Adjusted Odds Ratio.

## 4 Discussion

The study observed changes in KAP for the intervention group. These findings agreed with those of previous school-based studies [[36], [37], [38]]. The baseline level for KAP was high for both groups, especially knowledge about the threat of the disease, *Aedes* breeding sites and preventive and control measures for dengue. This result reflects the efficacy of the existing health educational programme or live messages using other routes, such as social media. Several studies reported the regular exposure of high school students in Myanmar to social media [39,40]. Nowadays, technology has become the main channel for delivering health information [23]. Moreover, electronic health and mobile health are used for healthcare delivery, disease surveillance, health education and health promotion [24,25]. Such online open sources may be related to the health education background in a growing city such as Yangon.

The high baseline scores for knowledge and awareness about dengue as a life-threatening disease (e.g. ‘It is a deadly disease’ and ‘It occurs every season’) were detected for both groups. According to the health belief model, awareness of the threat of a disease is a key factor that leads to the active seeking of information, which may lead to other significant changes in practices [36,26]. Nearly all issues related to knowledge and attitude significantly increased for the intervention group after the training. Interestingly, a common serious clinical sign, bleeding, was less recognised, which may be related to the Burmese language. In Myanmar, dengue haemorrhagic fever is called *Thawe Lon Tut Kwe*, which means flu disease and leaves out the haemorrhage part. The World Health Organization issued best practices for naming new infectious diseases, which were frequently given by people outside the scientific community, thus exerting unintended negative impacts and unnecessarily leading to untoward effects [27].

Furthermore, the students misunderstood that rice fields and dirty water were common outdoor breeding sites prior to the programme; however, this perception changed for the intervention group. These findings were similar to those of a study in Malaysia, where nearly half of the students misunderstood that *Aedes* mosquitoes prefer to lay eggs in dirty water [36]. Moreover, confusion may have emerged regarding other mosquito-borne diseases, such as Japanese encephalitis, malaria and lymphatic filariasis. Thus, similar training programmes should provide knowledge about other epidemic vector-borne diseases so as to fit with the integrated vector control programmes of the WHO as well as the local context for disease prevention and control measures [28].

Overall attitude exhibited changes after the intervention. However, only the attitude towards the negative perception of harmful mosquito coils was retained by majority of the students after the intervention. The misunderstanding about the ‘toxicity of temephos’ was high at baseline for both groups but changed to a positive attitude in the intervention group after the training. Regarding the perception of temephos, Legorreta-Soberanis et al. [29] conducted a community-based study in Mexico and reported that 69% of people believed that bathing and drinking water that contain temephos posed health risks. Thus, information provided by health care personnel, community leaders and related personnel in this area should be explored.

Although knowledge and awareness of dengue increased after training, the extent to which knowledge can be translated into practices and how the practice will in fact influence the larva population remain unclear. Despite the association between knowledge and preventive behaviours towards dengue in several studies [36,30,31], knowledge alone could not directly translate into practices or predict practices [[32], [33], [34], [35]]. Koenraadt et al. [41] reported that the gap between knowledge and practice is an important challenge for dengue control and a dynamic target of the reduction of the *A. aegypti* population. The current study found that self-reported practices significantly increased for the intervention group; however, such practices were observed by less than 50% of households. Similar findings were found for Myanmar wherein low scores for practices were observed among caregivers with high levels of knowledge [42]. Khun and Manderson et al. [18] suggested that a long-term school-based dengue programme was required to ensure the translation of knowledge into practice. Moreover, selective health messages that are relevant to daily practices should be identified to change knowledge into practice for dengue prevention and control.

Despite the increased knowledge and decreased negative attitude towards temephos after training, the self-reported practices of the intervention group remained the same. The survey also found that only 50% of the households actually used temephos after the training. The acceptability of temephos is complicated, which was outside the scope of the study. Thus, we infer that this result is influenced by the misconception of temephos as a harmful chemical or simply the lack of knowledge of how to use it. Thavara et al. [43] reported that the refusal to use temephos may be owing to its unpleasant door, water turbidity and perceived safety risks. In Yangon, the responsibility for regular control practices, including the distribution of temephos, is shouldered by the local health department and community leaders [44] such that the residents assumed that these tasks were the responsibility of the authorities instead of their own. Apart from the issue of temephos utility, this study also demonstrated an increase in the percentage of weekly cleaning and covering of water containers for the intervention group. Nevertheless, less than half of the households actually practiced these measures. Phuanukoonnon et al. [45] found that the size, shape and location of water containers determine the success of these methods in Thailand.

Moreover, the current study did not report on larvivorous fish despite its advantages over other prevention and control methods because of its feasibility, low cost and being environmentally friendly. This concept may be due to culture, because owning a fish tank or pond in the household (which is common in Thailand) is uncommon in Yangon. The larval survey form used in this study was created by researchers based on the Thai culture and lifestyle (Mahidol University). Thus, adapting the tool to the Myanmar culture would have been favourable. A qualitative study should also be conducted prior to the tool creation for the survey among health authorities, community leaders and other stakeholders.

Suwanbamrung et al. [20] conducted a school-based participatory education programme in Thailand, reporting an increase in knowledge on dengue prevention and control, which was associated with a reduction in larval indices in the school and the households of the students. The current study obtained similar findings, such as the decreased larval indices for the intervention group after training. Although the level of larval indices apparently decreased, the CI remained slightly higher than the standard criteria set by the WHO [46] as well as the applied larval indices of the MOHS in Myanmar (HI > 10%, BI > 20%) [44]. However, other factors may influence this efficacy because the larval indices also decreased for the control group. The first survey was conducted during the wet season with slightly higher number of water containers compared with the second survey, which was conducted during the dry season. Many studies reported that seasonal fluctuations influence the rate *Aedes*-positive containers in residential and public areas owing to differences in rainfall, temperature, evaporation and humidity between the two seasons [47,48]. During the dry season, people may have consumed all stored water such that the majority of water containers are empty (during the second survey), which may also influence the larval indices. However, with the disproportionate change in containers (slightly decreased number of containers: marked decreased of larval indices), this hypothesis may not be the only reason for the marked decrease in larval indices during the post intervention survey in both groups.

This study also explored the effectiveness of the training by testing the correlation between KAP levels and larval positivity. Only high levels of knowledge and high scores for self-reported practices were significantly related to less larval positivity. Previous studies supported that knowledge gain may lead to successful behaviour modification. For example, Castro et al. [49] proposed that people with better access to information about dengue may assume a better understanding of the disease, which could lead to practices related to dengue. Moreover, many studies supported the direct link between knowledge and the adoption of best practices for the reduction of the breeding sites of *A. aegypti* after health education activities [31,50]. One of the possible reasons for these strong associations in the current study were the self-experimental training, which enabled the students to actually practice and efficiently translate it to family members compared with given simple reading materials. This result is similar to that of a study conducted on high school students that found that they are capable of communicating well with family members than primary school students in Thailand [51].

The most important goal of school health education programmes should be sustainable behavioural changes [52]. Local health departments and school health teachers should provide health education on dengue at least once a year at the beginning of the monsoon season in Myanmar. However, such programmes, which are provided through conventional lectures and involve printed materials, are not a major part of the curriculum. Thus, identifying a suitable tool for knowledge transfer to students is key for motivating families to increase their awareness and participating in dengue prevention practices. Students are interested in ludic strategies and practical activities, which was demonstrated by the significantly high KAP scores obtained by studies in Brazil [53] and the Philippines [50]. Evidently, these mixed-experimental methods in the training could demonstrate its effectiveness of it. A study provided evidence of the effectiveness of mixed-experiment teaching in health about obesity prevention among school children [54]. Thus, the current study proposes that learning by doing may motivate the students compared with conventional lectures. Nevertheless, mixed-experimental training is unique to each disease and is difficult to implement and sustain because it is time consuming and has limited resources despite the strong evidence of its efficiency at the community level [55]. Conversely, the new life style for sustainable learning through the use of social media and online platforms could be an alternative.

In 2015, UNESCO, Ericsson, the UK Department of International Development (DFID) and Ministry of Education initiated the Information Communication Technology (ICT) for Education Project across rural schools in Myanmar to create innovative teaching approaches and spread knowledge among the teachers and pupils [56]. Evidently, youth from resource-poor communities exhibited greater online engagement and positive changes in self-reported health behaviour for prevention against an arboviral disease in the Dominican Republic [57]. Lwin et al. [58] revealed that the youngest age group reported the highest levels of perceived severity and susceptibility to dengue and were well equipped with media exposure through the Internet and social media. Against this background, schools may need to emphasise a new paradigm for sustained educational training based on online learning programmes and should be adept to access social media and obtain credit for science subjects for lifelong learning.

## 5 Conclusion

The student training programme on dengue fever among the high school students in the Yangon region exhibited short-term effects regarding knowledge, attitude and self-reported practices. Despite the significant increase in KAP levels, they could not be translated into family practices and significant changes in the larval indices. Thus, this study recommends the use of the mixed-experimental training for future studies. To achieve the goal of dengue prevention and control, vector control activities may not be adequate despite the significant effort to decrease the dengue burden, and other alternative methods may be required. Vaccine implementation was proposed and the acceptance of the vaccine in this population should be explored [59].

## 6 Limitation

The main limitation of this study is that it was conducted only for 8 months. Moreover, assessment of the study outcomes was performed only two times, that is, 3 months before and after the training programme. Thus, a longer period is required to determine the long-term results.

## Author contribution statement

Soe Htet Aung: Conceived and designed the experiments; Performed the experiments; Analyzed and interpreted the data; Wrote the paper.

Suparat Phuanukoonnon: Conceived and designed the experiments; Analyzed and interpreted the data; Contributed reagents, materials, analysis tools or data; Wrote the paper.

Aye Mon Mon Kyaw, Saranath Lawpoolsri: Conceived and designed the experiments; Wrote the paper.

Patchara Sriwichai: Conceived and designed the experiments; Contributed reagents, materials, analysis tools or data; Wrote the paper.

Ngamphol Soonthornworasiri: Conceived and designed the experiments; Analyzed and interpreted the data; Wrote the paper.

Podjanee Jittamala: Conceived and designed the experiments; Performed the experiments; Analyzed and interpreted the data; Contributed reagents, materials, analysis tools or data; Wrote the paper.

## Data availability statement

Data will be made available on request.

## Declaration of competing interest

The authors declare the following financial interests/personal relationships which may be considered as potential competing interests: Podjanee Jittamala reports article publishing charges and writing assistance were provided by Faculty of Tropical Medicine. Podjanee Jittamala reports a relationship with Mahidol University Faculty of Tropical Medicine that includes: employment, funding grants, and non-financial support.

## Figures and Tables

**figs1:**
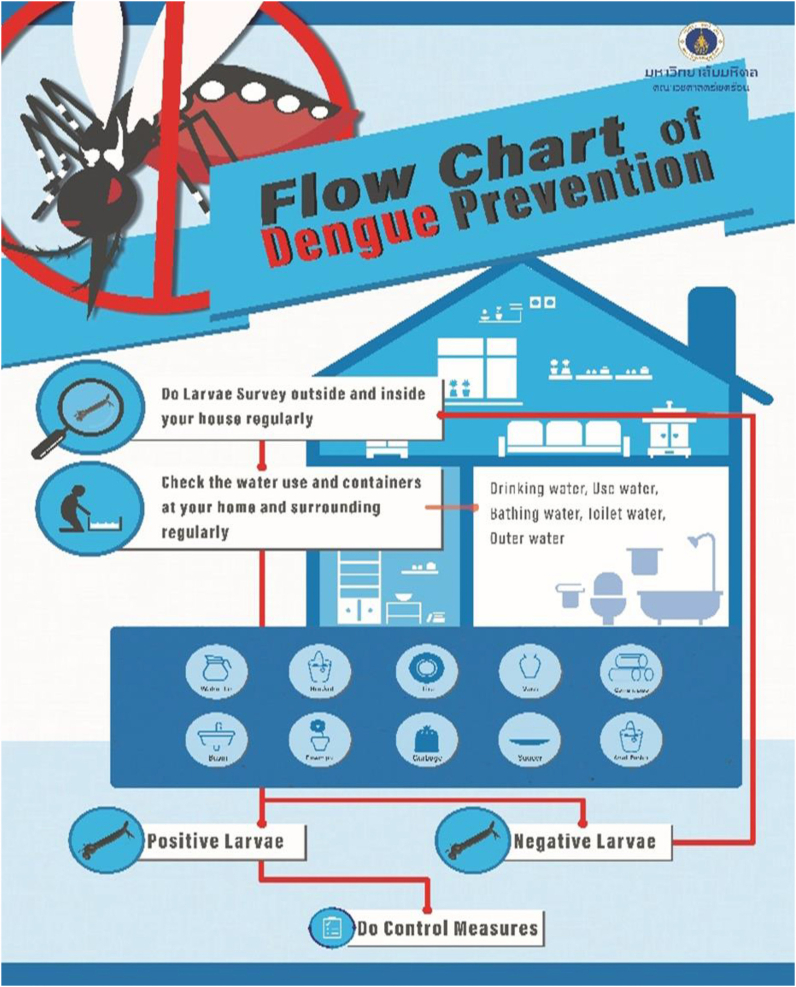


**figs2:**